# Drug-related problem and its predictors among pediatric patients with infectious diseases admitted to Jimma University Medical Center, Southwest Ethiopia: Prospective observational study

**DOI:** 10.1177/2050312120970734

**Published:** 2020-11-12

**Authors:** Desalegn Feyissa Mechessa, Dula Dessalegn, Tsegaye Melaku

**Affiliations:** 1 Department of Pharmacy, College of Medicine and Health Science, Mizan-Tepi University, Mizan-Aman, Ethiopia; 2 Department of Clinical Pharmacy, School of Pharmacy, Institute of Health, Jimma University, Jimma, Ethiopia

**Keywords:** Drug-related problem, pediatric, infectious disease, Ethiopia

## Abstract

**Background:**

Drug-related problem is any event involving drug therapy that may interfere in a patient’s desired clinical outcome. It has been pointed out that hospitalized pediatric patients are particularly prone to drug-related problems. Thus, this study aimed to assess drug-related problems and its predictors among pediatric patients diagnosed with infectious diseases admitted to Jimma University Medical Center, Southwest Ethiopia.

**Methodology:**

A prospective observational study was conducted among pediatric patients with infectious diseases admitted to the Jimma University Medical Center. Drug-related problems were classified based on Cipolle, Morley, and Strand’s drug-related problems classification method. The patient’s specific data were collected using a structured questionnaire. Data were entered into Epi data version 4.0.2 and then exported to statistical software package version 21.0 for analysis. To identify predictors of drug-related problems occurrence, multiple stepwise backward logistic regression analysis was done. Statistical significance was considered at a p-value < 0.05.

**Results:**

Of the total 304 participants, 226 (74.3%) of them had at least one drug-related problem during their hospital stay. A total of 356 drug-related problems were identified among 226 patients. Anti-infective medication was the major class of drug involved in drug-related problems. Noncompliance (28.65%) and dose too low (27.53%) were the most common type of drug-related problems identified. Presence of disease comorbidity (adjusted odds ratio = 3.39, 95% confidence interval = 1.89–6.08), polypharmacy (adjusted odds ratio = 3.16, 95% confidence interval = 1.61–6.20), and more than 6 days stay in hospital (adjusted odds ratio = 3.37, 95% confidence interval = 1.71–6.64) were independent predictors for the occurrence of drug-related problems..

**Conclusion:**

Drug-related problems were high among pediatric patients with infectious disease in the study setting. The presence of comorbidity, polypharmacy, and prolonged hospital stay were predictors of drug-related problems in this finding. Therefore, to prevent these problems, the collaboration of clinical pharmacists, pediatricians, and other health care professionals is needed during the provision of pharmaceutical care.

## Introduction

Infectious diseases are the major causes of death and morbidity among children worldwide, especially in developing countries.^1^ It is the most common medical condition of pediatric patients in Ethiopia.^2^ These infectious diseases are mainly managed by appropriate drug therapy. However, irrational prescribing, dispensing, and use of these therapies result in drug-related problems (DRPs).

DRP is an event or circumstance involving drug therapy that actually or potentially interferes with the desired health outcome. A potential DRP is not yet manifested, but if left unresolved, it may harm the patient. However, an actual DRP has resulted in clinical manifestations. DRP may arise at all stages of the medication use process from prescription to follow-up of the treatment.^3,4^

Pediatric patients are especial populations who need special attention in their drug therapy. However, they have been faced with many DRPs. This might be due to the differences in drug pharmacodynamics, pharmacokinetics, clinical heterogeneity, and a limited number of studies available concerning the safety and effectiveness of the drugs among these specific groups of population.^5,6^ The differences in drug pharmacokinetics and pharmacodynamics observed in children influence the choice of the drug, dose, dosage form, and dosing interval.^7^

Different DRP classification systems have been published in the literature. To date, there is no consensus and uniform method of classification of DRPs. However, according to Cipolle, Morley, and Strand, DRPs can be categorized into seven types: need additional drug therapy, unnecessary drug therapy, ineffective drug therapy, dose too low, dose too high, adverse drug reactions, and noncompliance.^8,9^

DRP causes significant mortality, morbidity, and also an economic crisis in the health care system. The estimated annual cost of drug-related morbidity and mortality resulting from nonoptimized medication therapy was $528.4 billion in 2016 US dollars, with a plausible range of $495.3–$672.7 billion.^10,11^ Besides, it also causes the hospital admission of pediatric patients in different countries. For instance, a study conducted in Canada and Australia showed that 8% and 4.3% of admission were related to DRPs, respectively.^12,13^ The major classes of drugs involved in the DRPs were anti-infectives.^14^

Studies showed that polypharmacy, type of medical conditions, type of admission, length of hospital stay, and number of medical conditions were the factors that were associated with DRP among pediatric patients.^15^–^17^

The magnitude of DRPs and its predictors was not studied among these populations in the study setting. Hence, this study aimed to assess DRPs and its predictors among pediatric patients diagnosed with infectious diseases admitted to Jimma University Medical Center (JUMC).

## Methods

### Study setting and period

The study was conducted from 1 April to 30 June 2018 in the pediatric ward of JUMC, which is located in Jimma town, Southwest Ethiopia, which is 352 km away from Addis Ababa, the capital city of Ethiopia. The hospital provides services for 9000 inpatients and 80,000 outpatients per year with a catchment population of approximately 15 million people. About 1623 patients were diagnosed with infectious disease in the pediatric ward of JUMC in 2017.

### Study design and population

Prospective observational study design was conducted on all pediatric patients with infectious diseases admitted to JUMC. All pediatric patients with infectious diseases admitted to JUMC during the study period were the source of the population, whereas all pediatric patients with infectious diseases admitted to JUMC during the study period and fulfilled the inclusion criteria were enrolled for the study.

#### Sample size determination and sampling technique

The sample size was determined based on single population proportion formula $n=({\left(Z_{\alpha /2}\right)}^{2}P(1-p))/d^{2}$ with the assumption of 95% confidence interval (CI), marginal error (d) of 5%, Z_α/2_ = 1.96, and P = 31.57% (prevalence of DRP among pediatric patients in Zewditu hospital, Ethiopia).^18^ Then, after using the correction formula and 10% non-response rate, the required total sample size was 304. All patients diagnosed with infectious disease admitted to the pediatric ward of JUMC during the data collection period and fulfilled the inclusion criteria were consecutively included in the study.

### Inclusions and exclusions criteria

Patients admitted to the pediatric ward of JUMC during the study period, age less than 16 years old, diagnosed with at least one infectious disease, and whose parent signed the informed consent were included in the study, whereas readmitted patients, a patient admitted to intensive care unit (ICU) ward and patients’ admission time less than 24 h, were excluded from the study.

### Study variables

In this study, the dependent variable was a DRP. The independent variables included the number of drugs used, comorbidity, number of infectious diseases, number of the disease condition, sex, age, weight, place of residence, type of admission, culture, and duration of hospital stay.

### Data collection method

Data were collected through medical record reviews and family/caregiver interviews daily for their drug-related need using a prepared structured questionnaire. The family/caregivers were interviewed to collect information related to adherence/nonadherence and reason for nonadherence. The data collection tool was prepared by reviewing different studies for important variables that were used to assess DRPs.^3,8,9^ The main content of the tool included sociodemographic characteristics, disease, and medication-related questionnaires. The patient care process was performed for each patient diagnosed with infectious disease until discharge.

### DRPs identification and classification

In this study, DRPs were classified according to Cipolle, Morley, and Strand’s DRPs method.^19^ The DRPs were identified by an independent team of experts after reviewing of patient’s medical record and were evaluated against different guidelines such as WHO (World Health Organization) 2016, Micromedex, Medscape, Nelson *Textbook of the Pediatrics* (20th edition), Naranjo scale, Ethiopian pediatric hospital care 2016 and different therapeutic guidelines for their appropriateness in the order of indication, effectiveness, safety, and drug interaction. The experts used pharmacotherapy work-up during the identification of DRPs. The recommendations were done by a team of experts and forwarded to physicians and/or other health care providers during morning sessions and major rounds. The identified DRPs were classified as unnecessary drug therapy, needs additional drug therapy, ineffective drug therapy, dosage too low, adverse drug reaction, dosage too high, and noncompliance.

### Data management and quality assurance

Data were collected by four pharmacists (BSc holders) and supervised by one clinical pharmacist daily. One day training was given for data collectors and supervisor. The pilot test was conducted on 16 (5%) patients diagnosed with infectious disease in the pediatric ward of Shenan Gibe hospital. The collected data were cleared and checked every day for completeness and consistency. The data were entered into Epi data manager version 4.0.2 and double entry verification was made. Then, data were exported to SPSS version 21.0 for analysis.

### Statistical analysis

The data were explored to check outliers, missing data, and assumptions. During analysis, frequencies and percentages were used to describe categorical variables while means and standard deviations were used to describe continuous variables. Bivariate logistic regression was run for all independent variables to assess the association between the study outcomes and the independent variable. Variables with p-value < 0.25 in the bivariate analysis were included for multivariable logistic model to identify the predictors of DRP. Statistical significance was considered at a p-value < 0.05.

### Ethics consideration

The ethical clearance was obtained from the institutional review board (IRB) of Jimma University. Then, the letter was given to the chief executive officer of the hospital and head of the pediatric department. The written informed consent was obtained from legally authorized representatives (caregiver/parents) before the study. The name and address of the patient were excluded from the data collection format to ensure the patient confidentiality.

### Operational definition

- *DRP* is an event or circumstance involving drug therapy that actually or potentially interferes with the desired health outcome.^3^
- *Polypharmacy* is defined as the concomitant use of five or more prescription medications.^20^
- The *patient cares process* is collecting subjective and objective information about the patient; assessing the collected data to identify problems and set priorities; creating an individualized care plan that is evidence-based and cost-effective; implementing the care plan; and monitoring the patients during their hospital stay.^21^
- *Pharmacotherapy work-up* is the structured, rational thought process for making clinical decisions in pharmaceutical care.^22^
- *Comorbidity* is a medical condition diagnosed other than infectious diseases in the pediatric ward during the study period.
- *Unnecessary drug therapy* is a DRP that occurs when there is no valid medical indication for the drug at the time, multiple drug products are used when only single-drug therapy is appropriate, or the condition is best treated with nondrug therapy.^8^
- *Needs additional drug therapy* is a DRP that occurs when there is a medical condition needing new drug therapy, preventive therapy is needed to reduce the risk of developing a new condition, or a medical condition requires combination therapy for better efficacy.^8^
- *Ineffective drug therapy* is a DRP where the drug is not the most effective for the medical problem, the drug product is not effective for the medical condition, the condition is refractory to the drug product being used, or the dosage form is inappropriate.^8^
- *Dosage too low* is a DRP that occurs when the dose is too low to produce the desired outcome, the dosage interval is too infrequent, a drug interaction reduces the amount of active drug available, or the duration of therapy is too short.^8^
- *Dosage too high* is a DRP where the dose is too high or the dosing frequency is too short or the duration of therapy is too long for the patient, a drug interaction causes a toxic reaction to the drug product, or the dose was administered too rapidly.^8^
- *Adverse drug reaction* is a DRP where the drug product causes an undesirable reaction that is not dose-related, a safer drug is needed because of patient risk factors, a drug interaction causes an undesirable reaction that is not dose-related, or the regimen was administered or changed too rapidly.^8^
- *Noncompliance* is a DRP that occurs when the patient does not understand the instructions, the patient prefers not to take or forgets to take the medication, the cost of the drug product is not affordable for the patient, the patient cannot swallow, or the drug product is not available for the patient.^8^

## Results

### Clinical and sociodemographic characteristics of the study participants

Among 304 participants included in the study, 171 (56.3%) were male. One hundred sixteen (38.2%) of the study participants were infants. Nearly two-thirds of participants (63.8%) were residing in a rural area. The mean (±SD) weight of patients was 11.06 (±9 kg) (Table 1). From a total of 304 study participants, two-thirds (67.1%) of them had comorbidities and about 42% of patients had stayed within 6–10 days in the hospital with the mean duration of 8.98 ± 5.00. Two hundred twelve (69.7%) were admitted to the pediatric ward of JUMC by transferring from different health facilities. Nearly half of the study participants (47.7%) had a single infectious disease. One hundred twenty-seven (41.77%) of them had polypharmacy (Table 2).

**Table 1.** Sociodemographic characteristics of study participants in JUMC from 1 April to 30 June 2018.

Variables	Frequency (%) (N = 304)
Sex	
Male	171 (56.3)
Female	133 (43.7)
Age	
Neonate (birth to 28 days)	42 (13.8)
Infant (29 days to ⩽1 year)	116 (38.2)
Toddler (>1 to ⩽3 years)	45 (14.8)
Preschool (>3 to ⩽5 years)	36 (11.8)
School-age (>5 to ⩽10 years)	38 (12.5)
Adolescent (>10 to ⩽16 years)	27 (8.9)
Weight (kg)	
<5	92 (30.3)
5–9.9	91 (29.9)
10–14.9	45 (14.8)
15–19.9	25 (8.2)
20–24.9	19 (6.3)
⩾25	32 (10.5)
Place of residence	
Urban	110 (36.2)
Rural	194 (63.8)

**Table 2.** Clinical characteristics of study participants in the pediatric ward of JUMC from 1 April to 30 June 2018.

Variables	Frequency (%) (N = 304)
Comorbidity^a^	
Yes	204 (67.1)
No	100 (32.9)
Duration of hospital stay	
⩽5 days	76 (25)
6–10 days	128 (42.1)
⩾11 days	100 (32.9)
Number of infectious diseases	
1	145 (47.7)
2	116 (38.2)
⩾3	43 (14.1)
Culture done	
Yes	139 (45.7)
No	165 (54.3)
Number of the disease condition	
1	49 (16.1)
2	93 (30.6)
3	88 (28.9)
⩾4	74 (24.3)
Type of admission	
New	92 (30.3)
Transferred	212 (69.7)
Polypharmacy	
Yes	127 (41.77)
No	177 (58.33)

a Severe acute malnutrition, anemia, asthma, nephrotic syndrome, and so on.

### The types, causes, and prevalence of DRPs

**Figure 1.**
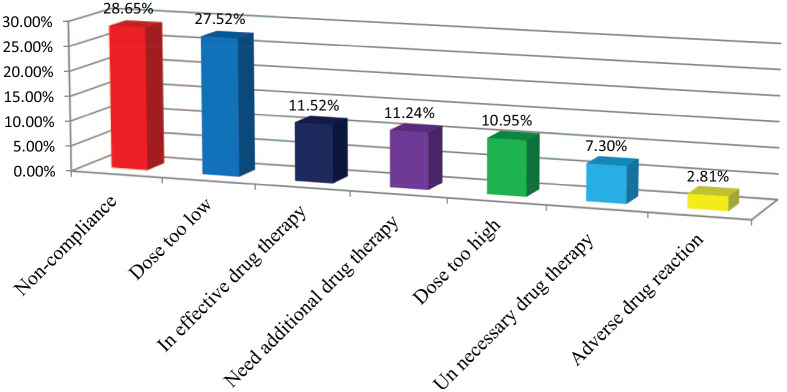
Types of drug-related problems identified among study participants in the pediatric ward of JUMC from 1 April to 30 June 2018.

From a total of 304 patients, 226 patients experienced DRPs, with an overall prevalence of 74.3%. The most common DRPs identified were noncompliance (28.65%), followed by dose too low (27.52%) (Figure 1). The causes of each DRP were explained in detail in Table 3.

**Table 3.** The common causes of DRPs identified among study participants in the pediatric ward of JUMC from 1 April to 30 June 2018.

DRP category and cause	Frequency (%)
Noncompliance	102 (28.65)
The caregiver does not understand the instructions	31 (30.4)
The drug product is too expensive for the patient	29 (28.43)
Omission (vein is not visible)	19 (18.63)
Caregiver forgets to give the medication	12 (11.76)
The drug product is not available for the patient	11 (10.78)
Dose too low	98 (27.53)
The dose is too low to produce the desired response	54 (55.10)
Dosage interval is too infrequent	28 (28.57)
Drug interaction	13 (13.27)
Duration of drug therapy is too short	3 (3.06)
Ineffective drug therapy	41 (11.52)
The drug product is not an effective product	28 (68.29)
The dosage form of the drug product is inappropriate	6 (14.63)
Condition is refractory to the drug product	4 (9.76)
Other^a^	3 (7.32)
Need additional drug therapy	40 (11.23)
A medical condition requires the initiation of drug	31 (77.5)
Preventive drug therapy is required	5 (12.5)
To attain a synergistic effect	4 (10)
Unnecessary	26 (7.3)
Multiple drug products are being used	17 (65.4)
No valid medical indication	9 (34.6)
Dose too high	39 (10.96)
The dose is too high	16 (41.02)
Drug interaction	15 (38.46)
Duration of drug therapy is long	4 (10.25)
The dosing frequency is too short	4 (10.25)
Adverse drug reaction	10 (2.81)
Drug product causes an allergic reaction	4 (40)
The drug product is contraindicated due to risk factors	6 (60)

DRP: drug-related problem.

a Microorganisms develop resistance to drug product.

### Drugs involved in DRPs

The major class of drugs involved in DRPs was systemic anti-infectives (271 (76.12%)), followed by central nervous system medicine (16 (4.49%)) (Table 4).

**Table 4.** The class of drugs involved in drug-related problem among study participants in the pediatric ward of JUMC from 1 April to 30 June 2018.

Class	Frequency (%) (N = 304)
Systemic anti-infective medicines	271 (76.12)
Central nervous system medicines	24 (6.74)
Gastrointestinal medicines	15 (4.21)
Cardiovascular medicines	11 (3.09)
Dermatological medicine	8 (2.24)
Medicines used in endocrine disorder	6 (1.69)
Respiratory medicines	5 (1.40)
Others^a^	16 (4.50)

a Electrolyte and acid-base balance correcting drugs, vitamins, medicines affecting the blood, and ophthalmic agents.

### The type of interventions provided

**Figure 2.**
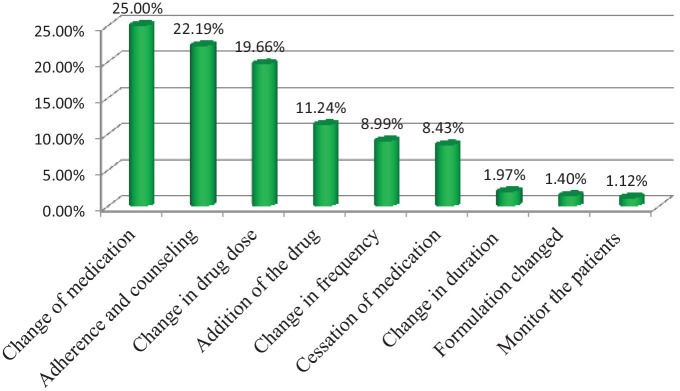
Type of intervention provided for study participants in the pediatric ward of JUMC from 1 April to 30 June 2018.

The major type of interventions provided was a change of the medication (89 (25%)), followed by adherence and counseling (79 (22.19%)) (Figure 2).

### Predictors of DRPs

On multivariate logistic regression analysis, comorbidity, polypharmacy, and prolonged hospital stay were found to be independent predictors of the occurrence of DRPs among study participants (Table 5).

**Table 5.** Multivariate logistic regression analysis of DRPs among study participants in the pediatric ward of JUMC, 2018.

Variables	COR (95% CI)	AOR (95% CI)	p-value
Duration of hospital stay			
⩽5 days	1	1	
6–10 days	3.70 (1.98–6.94)	3.37 (1.71–6.64)	0.0004*
⩾11 days	4.39 (2.20–8.75)	3.86 (1.84–8.08)	0.0003*
Polypharmacy			
No	1	1	
Yes	4.57 (2.42–8.62)	3.16 (1.61–6.20)	0.001*
Comorbidity			
No	1	1	
Yes	3.92 (2.29–6.74)	3.39 (1.89–6.08)	0.00004*
Number of infectious diseases			
1	1	1	
2	1.74 (0.99–3.06)	1.19 (0.61–2.32)	0.59
⩾ 3	2.95 (1.16–7.49)	1.18 (0.39–3.57)	0.76
Weight (kg)			
<5	0.59 (0.15–2.24)	0.66 (0.15–2.84)	0.58
5–9.9	0.58 (0.15–2.20)	0.82 (0.19–0.34)	0.79
10–14.9	0.46 (0.11–1.85)	0.60 (0.13–2.68)	0.50
15–19.9	0.75 (0.15–3.62)	0.82 (0.14–4.61)	0.82
20–24.9	1	1	
⩾25	0.27 (0.06–1.13)	0.33 (0.33–1.56)	0.16
Number of the diseases condition			
1	1	1	
2	2.68 (1.30–5.51)	0.95 (0.30–2.99)	0.93
3	4.35 (2.01– 9.40)	0.72 (0.14–3.54)	0.69
⩾ 4	6.67 (2.79–15.92)	0.75 (1.12–4.44)	0.75

DRP: drug-related problem; COR: crude odds ratio; CI: confidence interval; AOR: adjusted odds ratio.

* Statistically significant (p < 0.05).

## Discussion

The study of potential DRP in pediatric patients is very essential in the prevention of complications arising from DRPs.^23^ In this finding, the prevalence of DRPs was found to be 74.3%, which was higher than that of the study conducted in Hong Kong (21%) and Ethiopia (31.57%).^15,18^ This difference might be due to the difference in the hospital setting, the difference in DRPs classification used and the availability of trained prescribers, and clinical pharmacists in the pediatric ward.

The type and cause of DRPs were reported in this finding. The most common DRP identified was noncompliance (28.65%) which was comparable with the study done in Côte d’Ivoire (24.1%).^24^ However, it was higher than the study done in Northeastern Ethiopia (20.2%).^25^ The difference might be in the study area, and the majority of the patients were prescribed with multiple drugs that may cause unaffordability and difficulty in understanding the instructions of the drugs. This collectively may contributed for noncompliance.

In the present study, dose too low was found to be 27.52% which was comparable with the study done in Egypt (21.09%).^26^ However, it was lower than the study done in the kingdom of Saudi Arabia (58.6%).^27^ In the present study, dose too high was found to be (10.95%) which was lower than the study done in Hong Kong (19.3%).^15^ This showed that inappropriate doses are more common in pediatrics which might be because of weight-based dose calculation, fractional dosing, and incorrect recording of patients’ weights and prescribing error.^28^ In the current study, adverse drug reaction was found to be 2.81% which was in line with the finding reported in Toronto (2.5%).^29^

In this study, need additional drug therapy was 11.24% which lower than the study done in Northeastern Ethiopia (25.2%).^25^ In our study, unnecessary drug therapy was 7.3% which was in line with the study done in Addis Ababa. However, this finding was two times higher than the study done in the United Kingdom and Saudi Arabia (3.8%). This indicated that duplicate drug therapy was common in the study area that contributes to the patient to pay the extra cost and expose them for drug interaction. Therefore, the prevention of duplicate drug therapy will contribute to cost-saving among hospitalized patients.

Pharmacists have a crucial role in the health care system. Studies showed that the involvement of pharmacist in pediatric care can significantly help to identify, resolve, and prevent DRPs.^29,30^ In the present finding, the most common type of intervention provided was the change of the medication (25%) which was similar to a study done in India where changing of the medication is the most common type of intervention provided.^23^ In the present study, adherence and counseling provided for patients was found to be 22.19%. This was higher than the finding reported by Rashed et al.^15^ The discrepancy might be due to the dosing problem was the most common type of DRP, whereas noncompliance was the most common DRP in this finding.

The present finding showed that polypharmacy was found to be independent predictors of DRP. Similarly, a study conducted in Zewditu hospital,^18^ Hong Kong,^15^ and the United Kingdom and Saudi Arabia^16^ revealed that polypharmacy was the predictor of DRPs in pediatrics.

In this finding, prolonged hospital stay was the predictor of DRPs. Similarly, the finding reported by Eshetie et al.^17^ and Dedefo et al.^31^ supported that prolonged hospital stay was the risk factor for the occurrence of DRP. The possible reason could be the more the patient stayed in the hospital, the more likely the patient had a chance to acquire new infections such as hospital-acquired infection and health care–associated infection. These infectious diseases need new and more complex medications which further contributed to occurrence of DRP.

In the current study, the presence of comorbidity was one of the independent predictors for DRP. This was also supported by the finding reported by Zed et al.^32^ The reason might be the presence of comorbidity influences the desired outcome of other diseases by increasing number of drugs and causing disease–disease interaction, drug–drug interaction, and drug–disease interaction, which collectively results in increased likely hood of experiencing DRPs in the study patients.

### Limitations of the study

The study did not assess the severity level of DRPs such as mild, moderate, and severe. Besides, it did not assess the outcome of intervention provided and also the study was conducted at a single institution.

## Conclusion

The present finding showed that the majority of study participants had at least one DRP during their hospital stay. The most frequently identified DRP was noncompliance, followed by dose too low. The finding revealed that the presence of comorbidity, polypharmacy, and prolonged hospital stays were independent predictors of DRPs. Therefore, to minimize these problems, clinical pharmacists, pediatricians, and other health care professionals have to work in collaboration during the provision of pharmaceutical care. Finally, the authors recommend that researchers have to conduct research on clinical, economical, and humanistic impact of DRP among pediatric patients diagnosed with infectious disease admitted in pediatric ward.

## Supplemental Material

questionnaries_and_supplementry_data – Supplemental material for Drug-related problem and its predictors among pediatric patients with infectious diseases admitted to Jimma University Medical Center, Southwest Ethiopia: Prospective observational study Click here for additional data file. Supplemental material, questionnaries_and_supplementry_data for Drug-related problem and its predictors among pediatric patients with infectious diseases admitted to Jimma University Medical Center, Southwest Ethiopia: Prospective observational study by Desalegn Feyissa Mechessa, Dula Dessalegn and Tsegaye Melaku in SAGE Open Medicine
