# Supplementary material for: Drug-related problem and its predictors among pediatric patients with infectious diseases admitted to Jimma University Medical Center, Southwest Ethiopia: Prospective observational study
Source: SAGE Open Med. 2020 Nov 12;8:2050312120970734. doi: 10.1177/2050312120970734 (PMC7675898; doi:10.1177/2050312120970734)
Supplement: questionnaries_and_supplementry_data – Supplemental material for Drug-related problem and its predictors among pediatric patients with infectious diseases admitted to Jimma University Medical Center, Southwest Ethiopia: Prospective observational study [file questionnaries_and_supplementry_data.pdf]

ANEX I: Patient Written informed Consent Form in English version

Dear/ Sir/Madam,

My name is Desalegn Feyissa. I am going to conduct a research on Drug related problems and its predictors among pediatrics aamong patients diagnosed with infectious disease admitted to pediatric ward of Jimma University medical center. Therefore, for the success of this research your child's medical chart and your response to interview are paramount. Any information of your child's from you and medical records would be completely confidential to the research and the data are stored without his/her name and only used for the purpose of this study. None of this would affect the care your childs' receive from JUMC, rather it will contributes in managing your childs' medical problem and also help in future planning for the hospital care. No identifying names or characteristics will go into my report, so you may share your thoughts openly about your child's. Please ask me to stop as we go through the information and I will take time to explain. I would be grateful if you could sign the attached form to say you have no objections to our accessing any records and interviewing you about your child. Would you be willing to assist me by having a 15-20 minutes' interview with me? If the interviewee responds "Yes" please proceed and let him/her to sign or if replies "No" gratitude him/her and quit the interview. If you have any questions concerning the study, please call Desalegn Feyissa (PI) (+251) 917-12 7556.

Signature of respondent ___________________________

Date: ____________________ (Day/month/year)

Principal Investigator: Desalegn Feyissa

Jimma University, IHS, School of Pharmacy, Department of Clinical Pharmacy

Email: sinaawayya@gmail.com

ANNEX II: DATA COLLECTION FORM ENGLISH VERSION

Data collection format for research paper entitled with “Drug related problem and its predictors among pediatrics patients with infectious disease admitted to Jimma university medical center from April 1,2018 to June 30,2018, Jimma, South west Ethiopia”.

Part I. Patient Socio-demographic characteristics

Age____ Card no________ Height (cm) ______Body surface area (BSA) ______
Sex____ Weight (kg) _____ Place of residence: Urban_____ Rural_____

Part II: Patient clinical information:

2.1 Diagnosis(infectious disease(ICD)___________

Date	diagnosis

2.2 Is there any comorbid condition? Yes…….. No……

2.3 If yes to question number 2.2 list comorbidities identified

a) ______________ c)__________________________
b) ______________ d)__________________________

2.4 Duration of hospital stays______

2.5 Type of admission: New______ Transferred_________

Part III. Patients’ medication information

1. Did your Childs’ take any medication before admission to this hospital? Yes___ No____
2. If yes for question number 3.1 list those medication

a) ___________ e)______________
b) ___________ f)______________
c) ___________ g)_______________

3. Medication given for patients’ from date of admission to discharge

Drug Name/strength/dose/route	Prescribed schedule	Duration Start date-stop date	Indication	Remark

4 Number of drugs prescribed per patient:____________

Part IV. Drug related problem identified

I. **Logical questions to identify whether or not the patient is experiencing a drug related problem**

1. Is there a need for additional drug therapy? Yes___ No____
2. If yes for no.1 what is the reason for additional therapy need?

a) A medical condition requires the initiation of drug therapy.

b) Preventive drug therapy is required to reduce the risk of developing a new condition.

c) To attain synergistic effect or additive effect

d) Others (specify)_____

3. If " yes" for number,1 please list those medical problems needing additional medication

Date	Indication	Drug regimen with the problem	cause

4. Is there any unnecessary drug therapy for the patient? Yes___ No____
5. If yes for number.4 what are the reasons for unnecessary drug therapy?

a) There is no valid medical indication for the drug therapy at this time.

b) Multiple drug products are being used for a condition that requires single drug therapy.

c) The medical condition is more appropriately treated with non-drug therapy.

d) Drug therapy is being taken to treat an avoidable adverse reaction associated with another medication.

e) Only life style can be used to control a medication

f) Other specify ____

6. If " yes" for number,4 please list those unnecessary prescribed medication and causes

Date	Indication	Drug regimen with the problem	cause

7. Is there any ineffective drug therapy used? Yes___ No____
8. If yes for number.7. What was the cause?

a) The drug is not the most effective for the medical problem.

b) The medical condition is refractory to the drug product.

c) The dosage form of the drug product is inappropriate.

d) The drug product is not an effective product for the indication being treated

e) Others (specify)_____

9. If yes for number 7 list the ineffective medication used?

Date	Indication	Drug regimen with the problem	cause

10. Is there any medication with too low dosage? Yes___ No____
11. If yes for number.10. What was the cause for the dosage too low?
    a) The dose is too low to produce the desired response.
    b) The dosage interval is too infrequent to produce the desired response.
    c) A drug interaction reduces the amount of active drug available.
    d) The duration of drug therapy is too short to produce the desired response.
    e) Other (specify)____
12. If yes for number 10 list those doses too low with their causes?

Date	Indication	Drug regimen with the problem	cause

13. Is there any medication with too high dosage? Yes___ No____
14. If Yes for question number.13 what is the cause for dosage to be high?
    a) Dose is too high.
    b) The dosing frequency is too short.
    c) The duration of drug therapy is long for a given condition.
    d) A drug interaction occurs resulting in a toxic reaction to the drug product.
    e) The dose of the drug was administered too rapidly
    f) Adjustment for renal impairment was not done
15. If yes for question number 13 .please list those with dose too high with their causes

Date	Indication	Drug regimen with the problem	cause

16. Is there any adverse drug reaction? Yes__ No____
17. If "yes" for number 16 what was the cause for the ADR?
    a) The drug product causes an undesirable reaction that is not dose-related.
    b) A safer drug product is required due to risk factors.
    c) A drug interaction causes an undesirable reaction that is not dose-related.
    d) The drug product causes an allergic reaction.
    e) The drug product is contraindicated due to risk factors
    f) Other (specify)____
18. If yes for question no 16 .please list those with ADR with their causes

Date	Indication	Drug regimen with the problem	cause

19. Is there any compliance problem? Yes__ No____
20. If "yes" for number 19 what was the cause for the non-compliance?
    a) The patient/caregiver does not understand the instructions.
    b) The patient/caregiver prefers not to take/give the medication.
    c) The patient/caregiver forgets to take/give the medication.
    d) The drug product is too expensive for the patient.
    e) The patient cannot swallow the drug product.
    f) The drug product is not available for the patient.
21. If yes for question number 19 .please list those drugs for which the patient non-compliant with its causes?

Date	Indication	Drug regimen with the problem	cause

Part V: Drugs involved in drug related problem

- Among the prescribed drugs specify which class and drug involved in drug related problem.

Date	Class of drug involved in DTP	Specific drug involved in DTP	comment

Part VI: Intervention taken by pharmacist

1. Type of intervention given

a) Addition of the drug______________ e) Change in drug dose_________
b) Adherence and counseling ___________ f) Change of medication ___________
c) Formulation changed ___________ g) other(specify)___________________
d) Cessation of medication__________

PLEASE! Fill the following information.

1. Name of data collector………………………………… Signature………….Date………..
2. Name of Supervisor ………………………………….. Signature………… Date………..

Annex III : Informed consent in Afan oromo version

Guca waliigaltee Afaan oromootin

Kabajamaa/ttuu Haadha/Abbaa hirmaataa/ttuu qorannoo kanaa. Ani Dassaalany Fayyissa yunivarsiitii Jimmaatti barataa kiliinikaal faarmaasii waggaa lammaffaa yoon ta'u, yeroo ammaa kana qorannoo waa'ee rakkoolee yaala qorichaan walqabatan giddu gala yaala fayyaa yunivarsiitii jimmaa kutaa daa'iman ciisanii yaalamanii keessa jiru irratti gaggeesufan jira. Kanaafuu galma gahiinsa qorannoo kanaatif deebiin afanii isin waa'ee yaala mucaa keesssanii naaf kennitan fi odeeffannoon kaardii yaala mucaa keessanii irra jiru baay'ee barbaachisaadha. Odeeffanoon isinirraa argamu maqaas ta'ee mallattoo eenyummaa mucaa keessanii kan hin qabnee fi iccitiidhan kan qabamudha. Hirmaachuu yookiin hirmaachuu dhabuun mucaa keessanii yaala fayyaa argatu/ttu irratti dhiibbaa hin qabu. Garuu, mucaan keessan furmaata rakkoolee mul'ateef kennamu irraa ni fayyadama/tti. Akkasumas qulqullina yaalaa gara fulduraatti hospitaalichaan kennamu foyyesuuf ni fayyada. Kanaafuu yaada keessan iftoominaan akka naaf laattan aferamtaniirtu.

Dabalataanis hirmaannaan kun guutumaan guututti fedhii iratti kan hundaa'eedha. Gaaffii deebisu hin barbaanne yoo jiraate, irra darbuu yookiin gaafachuu ni dandeessu. Yoo hirmaachuuf eeyyamamaa taatan guca kanarratti mallatteesun mirkaneessaa. **Eeyyee … lakki……..** yoo gaafii qabaatan bilbila kanaan naaf bilbilaa. Dassaalany Fayyissa (+251917127556).

Mallattoo gaafataa_______________

Mallattoo deebii kennaa______________

Guyyaa________________(guyyaa/ji'a/bara)

Dassaalany Fayyisaa

Jimma University, IHS, School of Pharmacy, Department of Clinical Pharmacy

Email: sinaawayya@gmail.com

Annex IV: <u>Data collection form in Afan oromo version</u>

Kutaa 1:Odeeffannoo jireenya hawaassummaa fi eeyyummaa Dhukkubsataa

Umrii_____Lakk Kaardii______ dheerina(cm)______ Bali'ina qaamaa______

Saala_____ Ulfaatina(kg)______ Bakka jireenyaa: Magaalaa____ Baaddiyaa_____

Kutaa 2: Gaaffilee lojikaawaa rakkolee yaala qorichaan walqabate dhukkubsataa mudate agarsiisu

1. Qoricha fudhachuu irratti rakkoon jiraa? Eeyyee__ lakki____
2. Deebiin Gaaffii lakk 19ffaa "eeyyee" yoo ta'e sababni isaa maali?
    a) Dhukkubsataan qajeelfama qorichaa hin hubanne
    b) Dhukkubsataan qoricha fudhachuu dhiisuu filate
    c) Dhukkubsataan qoricha fudhachuu hirranfate
    d) Dhkukubsataan qoricha liqimsuu hin danda'u/ttu
    e) Qorichi waan hin argamneef
    f) Dhukubsataan qoricha bitachuu waan hin dandeenyeef____
3. Deebiin Gaaffii lakk 19ffaa "eeyyee" yoo ta'e qorichi dhukkubsataan seeran hin fudhanne qabu fi sababa isaa tarreessi?

Guyyaa	dhibee kennameef	Qoricha seeran hin fudhatamne	sababa

Annex V : informed consent in Amharic version

የስምምነት ቅጽ

ጤና ይስጥልኝ? ስሜ ደሳለኝ ፈይሳ ይበላለሁኝ: የጅማ ዩኒቨሲቲ የሁለተኛ ዓመት የክሊኒካል ፈርማሲ ተማሪ ስሆን: በአሁኑ ሳዓት ከህክሚና መዳሃኒት ጋር የተያየዞ የሚመጡ ችግሮች በህፃነት ክፍል እየሰረዉ ይገኘለሁኝ። ስለዝህ ለዚህ ጥናት ስኬት ደግሞ የቃል ጥያቄ ስለ ልጃቸው ህክሚና የምትሰጡንነ ና በልጃቸው የህክሚና ካርድ ለይ የለዉ መረጀ እጅግ አስፈላጊ ነው። በዚህ ጥናት ዉስጥ ልጃቸዉ ማሰተፍ በልጀቸዉ ህክምና ለይ የምያመጣ አንድም ችግር የለም። ነገር ግን ለተፈጠረዉ ችግረ ለይ የምሰጠዉ መፍተሄ ተጠቃሚ ልሆን ይችላል/ትችላለች። የምትሰጡን መረጀ ስምም ሆነ የልጀቸዉ መንነት እይዝም/አያከቲትም: እንድሁም በምስጥር የምየዝ ይሆናል።

መሳተፍና አለመሰተፍ ልጀቸዉ ከምያገኘዉ ህክምና ላይ ምንም አይነት ጫና የለም: ነገር ግን ለወደፍት በ ሆስትታሉ የምሰጥ የህክምነ ጥራት የላቀ እንድሆን ለግበዓት ይሆናል። ስለዚህ ሀሳበችሁን በግልፅነት እንድትሰጡን ተጋብዛቿል። በተጨማሪም ተሳትፈችሁ ሙሉ በሙሉ ፍላጎት ላይ የተመሰረተ ነዉ: መመለስ የማትፈልጉ ጥያቄ ካለ መዝለል ወይም ደግሞ መጠየቅ ትችላለችዉ። ለመሳተፍ ፍቃደኛ ከሆኑት ቀጥለዉ በለዉ ቅፅ ላይ በፍርማ የረጋግጡልን።

ስለ ጥናቱ ጥይቄ ካለዎት ነጻ ሆነው ጠይቁኝ ጥናቱን የሚያጠናውን ሰው ከፈለጉ ከዚህ በታች ባሉት አድራሻዎእ ማግኘት ይችላሉ፡

የተሳታፊዉ ፊርማ፡ ____

የመረጃሰብሳቢፊርማ፡ ____

የዋናዉተመራማሪመረጃ፡-

ስም፡ ደሰለኝ ፈይሳ

ስሌክ፡ 0917127556

Email:sinaawayya@gmail.com

Jimma University, IHS, School of Pharmacy, Department of Clinical Pharmacy

Annex VI:Data collection form in Amharic version

ክፍል 1 : የተማሚዉ መረጃ

ዕድሜ------------ካርድ ቁጥር------------- ቁመት----------------የሰዉነት ሲፋት-----------

ፆታ-----------ክብደት--ከጂ----------የመኖራ ቦታ-ከተማ---------ገጠር-----------

ክፍል 2፡ ከህክሚና መዳሃኒት ጋር ተያይዞ ተማሚዉን ያ ጋጠመ ችግር የምያሳይ ሎጅካል ጥያቄ

1. መዳሃነት አወሳሰድ ለይ ችግር አለ? አዎ---------------------አይደለም-------------------

2. በተራ ቁጥር 1 ጥያቄ መልሶት አዎ ከሆነ ምክኒያቱ ምንድነዉ?

   ሀ.ታማሚዉ የመዳሃኒት አወሳሰድ ክትትል አላረገም

   ለ.ታማሚዉ መዳሃኒቱን አለመዉሰድ ስለመረጠ

   ሐ.ታማሚዉ መዳሃኒቱን መዉሰዱን ስለ ረሳ

   መ.ታማሚዉ መዳሃኒቱን መዋጥ ስለ አልቻለ

   ሰ. መዳሃኒቱ ስለ መይገኝ

   ረ. መዳሃኒቱን መግዛት ስለ ምይችል

3. በተራ ቁጥር 1 ጥያቄ መልሶት አዎ ከሆነ የጎኒዮች ጉዳት ያመጣ ና ታማሚ በአግባቡ ያልወሰደ መዳሃኒት ከነ ምክኒያቱ ይዘርዝሩት

ቀን	የተሰጠበት የበሽታ አይነት	በአግባቡ ያልተወሰደ መዳሃኒት	ምክኒያት

Annex VII: Category and common cause of Drug related problem

Need additional drug therapy

- A medical condition requires the initiation of drug therapy.
- Preventive drug therapy is required to reduce the risk of developing a new condition.
- A medical condition requires additional pharmacotherapy to attain synergistic effects

Unnecessary drug therapy

- There is no valid medical indication for the drug therapy at this time.
- Multiple drug products are being used for a condition that requires single drug therapy.
- The medical condition is more appropriately treated with non-drug therapy.
- Drug therapy is being taken to treat an avoidable adverse reaction associated with another medication.

Ineffective drug therapy

- The drug is not the most effective for the medical problem.
- The medical condition is refractory to the drug product.
- The dosage form of the drug product is inappropriate.
- The drug product is not an effective product for the indication being treated

Dosage too low

- The dose is too low to produce the desired response.
- The dosage interval is too infrequent to produce the desired response.
- A drug interaction reduces the amount of active drug available.
- The duration of drug therapy is too short to produce the desired response**.**

Adverse drug reaction

- The drug product causes an undesirable reaction that is not dose-related.
- A safer drug product is required due to risk factors.
- A drug interaction causes an undesirable reaction that is not dose-related.
- The dosage regimen was administered or changed too rapidly.
- The drug product causes an allergic reaction.
- The drug product is contraindicated due to risk factors

Dosage too high

- Dose is too high.

- The dosing frequency is too short.
- The duration of drug therapy is too long.
- A drug interaction occurs resulting in a toxic reaction to the drug product.
- The dose of the drug was administered too rapidly.

Noncompliance

- The patient/caregiver does not understand the instructions.
- The patient/caregiver prefers not to take/give the medication.
- The patient/caregiver forgets to take/give the medication.
- The drug product is too expensive for the patient.
- The patient cannot swallow or self-administer the drug product appropriately.
- The drug product is not available for the patient
